# The impact of previous ovarian surgery on ovarian reserve in patients with endometriosis

**DOI:** 10.1186/s12905-015-0230-1

**Published:** 2015-09-10

**Authors:** Hsin-Ju Chiang, Pin-Yao Lin, Fu-Jen Huang, Fu-Tsai Kung, Yu-Ju Lin, Pei-Hsun Sung, Kuo-Chung Lan

**Affiliations:** Department of Obstetrics and Gynecology, No.123, DAPI Rd. Niaosng Dist, Kaohsiung City, 83301 Taiwan R.O.C.; Division of Cardiology, Department of Internal Medicine, Kaohsiung Chang Gung Memorial Hospital and Chang Gung University College of Medicine, No.123, DAPI Rd. Niaosng Dist, Kaohsiung City, 83301 Taiwan R.O.C.

## Abstract

**Background:**

To investigate the impact of previous ovarian surgery on ovarian reserve in patients with endometriosis.

**Methods:**

A total of 829 female patients were recruited. Their medical records were reviewed retrospectively. Patients who had diagnoses of endometriosis or endometrioma were defined as the endometriosis group, and those without endometriosis were as the control group. We further divided these patients into four groups according to whether they had received ovarian surgeries before. Group 1: control group without previous surgery; Group 2: control group with previous surgery; Group 3: endometriosis group without previous surgery; Group 4: endometriosis group with previous surgery. The subgroups with endometrioma or not and different operative procedures were also analyzed. The parameters for comparison included age, body mass index, serum estradiol, follicle-stimulating hormone, luteinizing hormone, cancer antigen 125, and anti-Müllerian hormone (AMH) level.

**Results:**

The level of serum AMH was highest in group 1 and lowest in group 4. The decline was significant between group 1 and group 4 (*p* < 0.05). The serum AMH level was lower in group 4 than in group 3 but no significant difference. Serum estradiol level was significantly higher in group 3 than in group 2 (*p* < 0.05). Cancer antigen 125 levels were both significantly higher in group 3 and group 4 as compared with group 1 and group 2 (*p* < 0.05).

**Conclusions:**

Performing repeated ovarian surgery in patients with recurrent endometriosis needs careful consideration and adequate patient counselling because of the predictable deteriorating ovarian reserve.

## Background

Anti-Müllerian hormone (AMH) is secreted by pre-antral and antral follicles [1]. Unlike those predictors of ovarian reserve, e.g. basal follicle-stimulating hormone, estradiol, inhibin B, and antral follicle counts, a number of previous studies demonstrated AMH was a steadier marker to predict residual ovarian function (i.e. not being obviously influenced by menstruation) [2–5]. Changes in serum AMH level in patients with endometriosis have been well-documented. Remarkable declines in serum AMH were reported in patients with moderate to severe endometriosis, especially in those with bilateral endometriomas [6–12].

However, the impact of ovarian surgery on ovarian reserve in patients with endometriosis is still controversial. Some studies have reported decreased level of serum AMH after ovarian surgery [12–26], while others have reported steady serum AMH levels after ovarian surgery [27–30]. It is difficult to make definite conclusions due to selection bias of the study populations, surgeons’ expertise and skills, differences in the surgical techniques, and selected outcomes [31, 32]. In addition, the case numbers of these studies are also limited.

Patients with endometriosis usually receive ovarian surgery for disease treatment, symptomatic relief, and conception [33, 34]. At the out-patient department, clinicians often encounter this question: whether underlying endometriosis or previous ovarian surgery plays a more important role for the poor serum AMH level in patients with endometriosis. In addition, these patients with endometriosis may be referred from other hospitals, operated by different surgeons, and received different procedures. Therefore, the impact of previous ovarian surgery on ovarian reserve is difficult to estimate.

In this study, we tried to collect more numbers of patients with endometriosis than current publications in the out-patient setting, and performed a retrospective cohort study regardless of inter-surgeon variability to evaluate the impact of previous ovarian surgery on their ovarian reserve.

## Methods

### Patient enrollment and assignment

All of the patients were collected from December 2010 to June 2012 at the out-patient department of Obstetrics and Gynecology in Chang Gung Memorial Hospital, Kaohsiung Medical Center in Taiwan. To evaluate the impact of endometriosis and previous ovarian surgery on ovarian reserve, we focused on reproductive female patients. The inclusion criteria were: (1) female patients with regular menstrual cycles (interval within 25 to 35 days); and (2) female patients who had available blood samples of serum AMH at Chang Gung Memorial Hospital, Kaohsiung Medical Center. The exclusion criteria were: (1) male patients; (2) female patients who had not yet experienced menarche or who had reached menopause; and (3) female patients who were diagnosed with premature ovarian failure. All of their charts were retrospectively reviewed by a physician and a gynecologist, including out-patient department records, admission note, discharge note, transfer note, laboratory data, previous operative records, pelvic examination, and ultrasound findings. According to their medical records, the patients who had endometriosis or endometrioma were assigned into the endometriosis group, and those without endometriosis into the control group. In order to test our hypothesis that previous ovarian surgery may exacerbate ovarian reserve in patients with endometriosis, the endometriosis and control groups were further divided into four groups: group 1: patients without endometriosis had no history of ovarian surgery; group 2: patients without endometriosis had experienced ovarian survey before this clinical visiting; group 3: patients with endometriosis had no history of ovarian surgery; and group 4: patient with endometriosis had undergone ovarian surgery prior to this visiting.

In terms of the effects of endometrioma, we further divided the endometriosis group (group 3 and group 4) into subgroups with endometrioma or not, presented as following: subgroup A: endometriosis patients neither had history of ovarian surgery nor endometrioma; subgroup B: endometriosis patients without history of ovarian surgery had endometrioma; subgroup C: endometriosis patients had undergone ovarian surgery without endometrioma; subgroup D: endometriosis patients had both ovarian surgery history and endometrioma.

In our study, the minimal interval between the last surgery and clinical visiting were 3 months, and we thought it is reasonable to eradicate the immediate influence of serum AMH level after ovarian surgery. Different operative surgeons, procedures (laparotomy or laparoscopy, cystectomy, vaporization, combined cystectomy and vaporization, and only drainage) and lesions (ovarian endometrioma, endometriosis, teratoma or cyst) were all included in the previous ovarian surgery group. To discriminate the effect of different operative procedures in patients with endometriosis, the subgroups with different operative procedures, such as cystectomy, vaporization, combined cystectomy and vaporization, and only drainage were separately evaluated.

The Institutional Review Board of the Ethics Committee of Chang Gung Memorial Hospital approved this study (102-4853B). The need for consent was waived by the IRB.

### Laboratory assessments

All patients had available serum AMH data, and other parameters including age, body mass index (BMI), serum xestradiol (E2), follicle-stimulating hormone (FSH), luteinizing hormone (LH), and cancer antigen 125 (CA-125) were also assessed. All of the parameters were compared among these four groups.

The timing of blood sampling depended upon when the patients presented at the outpatient department and their recent menstruation. When the patients visited the outpatient department, serum AMH levels were obtained, regardless of the menstrual phase, by a commercial enzyme-linked immunosorbent assay kit (ELISA, Beckman Coulter, USA). The limit of the detectable level of AMH was 0.1 ng/ml, and the intra-assay and inter-assay coefficients of variation were 5.3 and 8.7 % respectively.

Other serum hormones were assessed in the early follicular phase of the menstrual cycle by commercially available immunoassay systems (ADVIA Centaur, Siemens, USA). The detection limits were 11.8 pg/ml for E2, 0.3 mIU/ml for FSH, and 0.07 mIU/ml for LH. CA-125 level was checked with the Architect CA 125 II assay (Abbott Diagnostics, USA), and the detection limit was 1.0 U/ml. All the serum samples were immediately separated by centrifugation at 4 °C for 15 min and stored at −80 °C until assay.

### Statistics

Statistical analyses were performed using SPSS (ver. 19.0; Statistical Package for Social Sciences, Inc., Chicago, IL, USA). Statistical evaluation of four groups was performed by one-way analysis of variance (ANOVA) with Bonferroni test for post hoc analysis. Continuous data are summarized as mean ± standard deviation. A probability values < 0.05 was considered statistically significant.

## Results

**Fig. 1:**
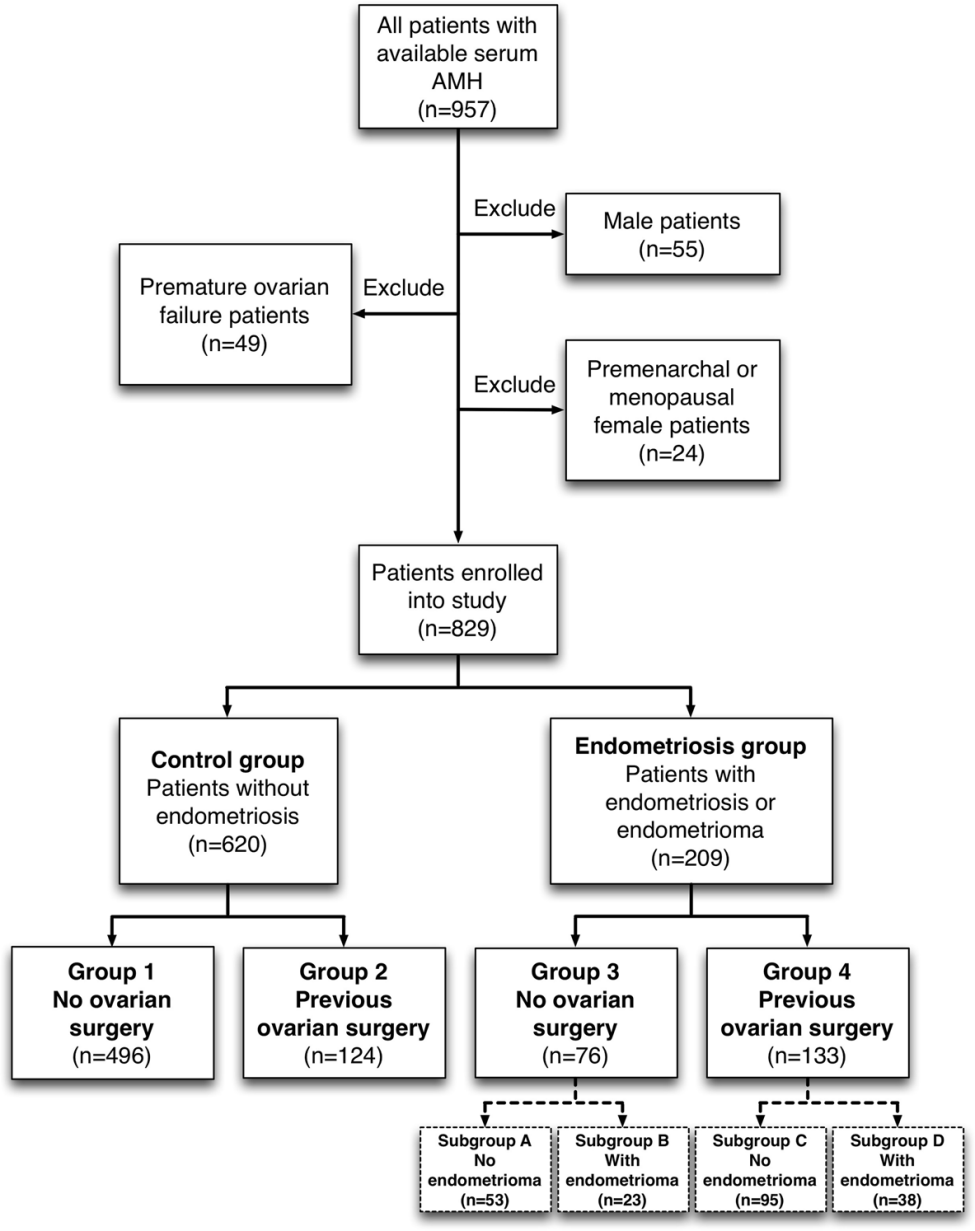
Patients enrollment flowchart

A total of 957 patients with available serum AMH data were collected. After 128 patients were excluded, the remaining 829 female patients were enrolled into this study. Among them, there were 209 (25.2 %) patients allocated in the endometriosis group, and 620 (74.8 %) patients in the control group. The endometriosis group and control group were further divided into four groups based on if previous ovarian surgery: group 1: the patients had no ovarian surgery in control group (*n* = 496), group 2: the patients with previous ovarian surgery in control group (*n* = 124), group 3: the patients had no ovarian surgery in endometriosis group (*n* = 76), group 4: the patients with previous ovarian surgery in endometriosis group (*n* = 133) (Fig. 1).

The period between blood sampling time and their previous surgery were ranged from three to twenty-four months, and the median of post-surgical months were ten months.

When comparing the parameters among the four groups, no significant differences were found in age, BMI, FSH, and LH (Table 1). However, a trend of decreasing serum AMH from group 1 to group 4 was noticeable. The highest serum AMH level was shown in group 1 and the lowest one was in group 4. The difference was statistically significant between them (4.2 ± 2.0 vs 2.3 ± 1.0 ng/ml, *p* < 0.05). Besides, the lower serum AMH level was noted in group 3 than in group 4, although not reaching significant difference.

**Table 1:** Clinical characteristics of 4 groups

	Group 1	Group 2	Group 3	Group 4	*p* value^a^
	Control: No ovarian surgery	Control: Previous ovarian surgery	Endometriosis: No ovarian surgery	Endometriosis: Previous ovarian surgery	
Number	496	124	76	133	
Age (years)	35.1 ± 5.9	36.1 ± 5.1	34.0 ± 5.0	35.3 ± 3.7	NS
BMI (kg/m^2^)	22.0 ± 3.8	22.6 ± 3.7	21.5 ± 3.4	22.1 ± 3.3	NS
E2 (pg/mL)	28.1 ± 13.5	20.2 ± 12.3	31.3 ± 17.0	24.4 ± 12.0	<0.05
FSH (mIU/mL)	6.2 ± 3.1	5.8 ± 3.0	6.0 ± 3.5	7.1 ± 3.5	NS
LH (mIU/mL)	5.5 ± 3.2	6.1 ± 3.6	3.7 ± 2.4	6.2 ± 3.8	NS
AMH (ng/mL)	4.2 ± 2.0	3.1 ± 2.0	3.1 ± 1.8	2.3 ± 1.0	<0.05
CA-125 (U/mL)	18.6 ± 12.9	22.2 ± 13.6	40.9 ± 27.4	45.3 ± 29.8	<0.05

*BMI* body mass index, *E2* serum estradiol, *FSH* follicle-stimulating hormone, *LH* luteinizing hormone, *AMH* anti-Müllerian hormone, *CA-125* cancer antigen 125, *NS* not significant (*p* > 0.05)

^a^Analysis of variance (ANOVA) with repeated measures

**Fig. 2:**
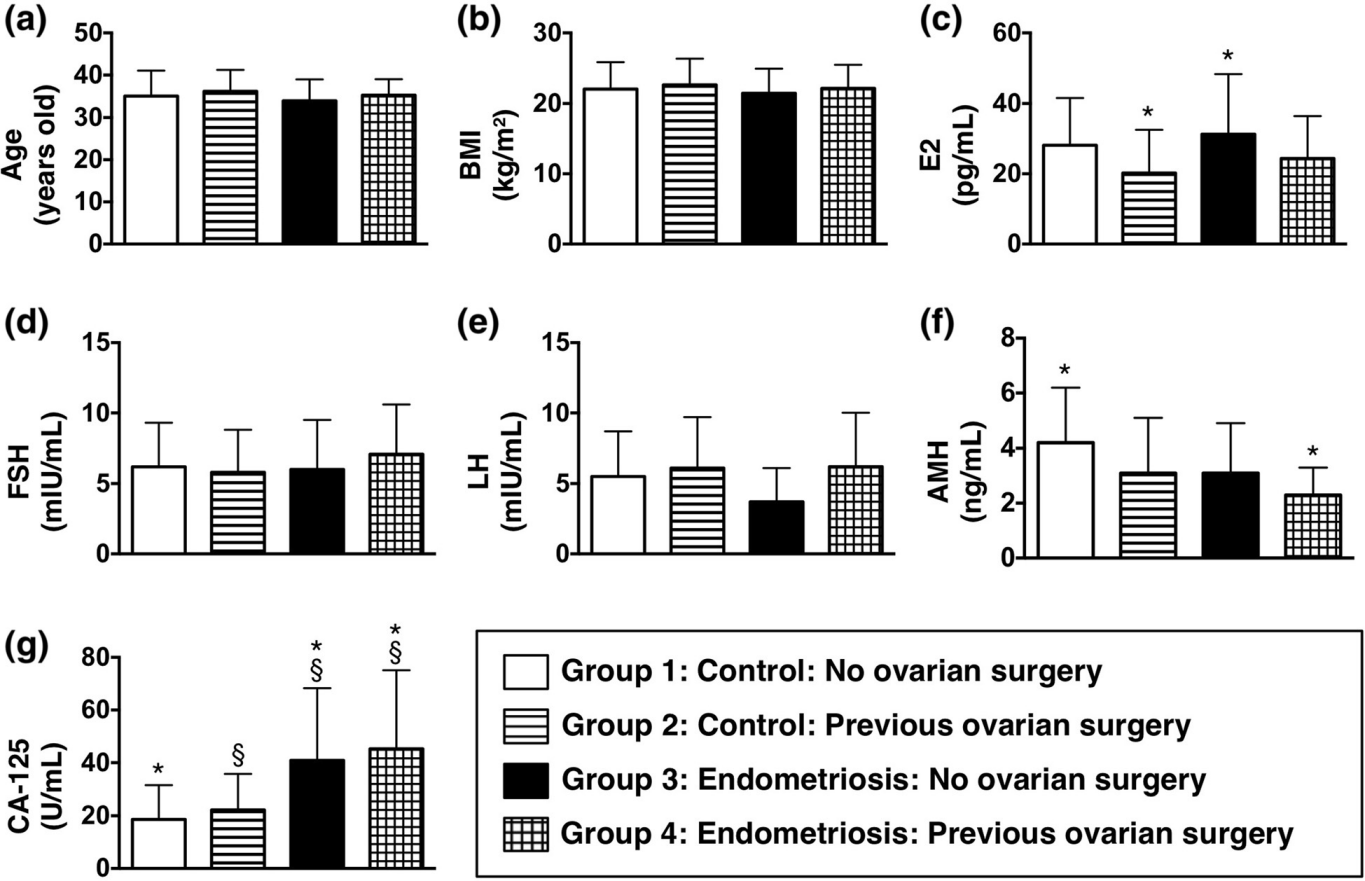
Parameters for comparison among 4 groups. (**a**) age (**b**) BMI: body mass index, (**c**) E2: serum estradiol, (**d**) FSH: follicle-stimulating hormone, (**e**) LH: luteinizing hormone, (**f**) AMH: anti-Müllerian hormone, (**g**) CA-125: cancer antigen 125. *, § = statistical significance (*p* < 0.05)

Serum E2 level was significantly higher in group 3 than in group 2 (31.3 ± 17.0 vs 20.2 ± 12.3 pg/ml, *p* < 0.05). CA-125 level were both significantly higher in group 3 and group 4 as compared with group 1 and group 2 (40.9 ± 27.4, 45.3 ± 29.8 vs 18.6 ± 12.9, 22.2 ± 13.6 ng/ml, *p* < 0.05) (Table 1) (Fig. 2).

The endometriosis group (group 3 and group 4) were further divided into 4 subgroups according to whether endometrioma was diagnosed or not: subgroup A: patients xhad history of neither ovarian surgery nor endometrioma (*n* = 53); subgroup B: patients without history of ovarian surgery had endometrioma (*n* = 23); subgroup C: patients undergoing ovarian surgery without endometrioma (*n* = 95); subgroup D: patients had both ovarian surgery history and endometrioma (*n* = 38) (Fig. 1). When comparing the parameters among them, there was no significant difference, although lower serum AMH and higher CA-125 were noted in endometrioma subgroup (Table 2).

**Table 2:** Clinical characteristics of endometriosis subgroups with endometrioma or not

	Subgroup A	Subgroup B	Subgroup C	Subgroup D	*p* value^a^
	No ovarian surgery	No ovarian surgery	Previous ovarian surgery	Previous ovarian surgery	
	No endometrioma	With endometrioma	No endometrioma	With endometrioma	
Number	53	23	95	38	
Age (years)	34.5 ± 5.5	32.8 ± 3.5	35.4 ± 3.6	34.9 ± 4.1	NS
BMI (kg/m^2^)	21.8 ± 3.6	20.9 ± 3.1	22.1 ± 3.6	22.2 ± 2.8	NS
E2 (pg/mL)	31.4 ± 25.7	31.0 ± 22.0	23.4 ± 18.9	27.4 ± 20.2	NS
FSH (mIU/mL)	6.3 ± 3.8	5.2 ± 2.1	7.2 ± 3.6	6.9 ± 3.3	NS
LH (mIU/mL)	3.8 ± 1.6	3.5 ± 1.4	6.5 ± 2.4	5.1 ± 2.9	NS
AMH (ng/mL)	3.3 ± 2.3	2.6 ± 2.0	2.3 ± 1.9	2.4 ± 1.6	NS
CA-125 (U/mL)	33.2 ± 23.1	50.7 ± 29.8	43.5 ± 27.3	48.6 ± 20.5	NS

*BMI* body mass index, *E2* serum estradiol, *FSH* follicle-stimulating hormone, *LH* luteinizing hormone, *AMH* anti-Müllerian hormone, *CA-125* cancer antigen 125, *NS* not significant (*p* > 0.05)

^a^Analysis of variance (ANOVA) with repeated measures

In different operative procedure subgroups of endometriosis patients, there were twenty patients received cystectomy, ninety-five patients received vaporization, fifteen patients received combined cystectomy and vaporization, and three patients received drainage only. The comparison of parameters among them showed no significant difference, although higher serum AMH and CA-125 were noted in combined cystectomy and vaporization subgroup (Table 3).

**Table 3:** Clinical characteristics of different surgical procedure subgroups in patients with endometriosis

	Cystectomy	Vaporization	Combined cystectomy and vaporization	Drainage	*p* value^a^
Number	20	95	15	3	
Age (years)	34.9 ± 4.5	35.4 ± 3.6	34.4 ± 3.7	37.7 ± 4.1	NS
BMI (kg/m^2^)	22.7 ± 2.0	22.1 ± 3.6	21.5 ± 3.7	22.1 ± 2.6	NS
E2 (pg/mL)	29.4 ± 14.6	23.4 ± 10.9	23.5 ± 19.3	29.4 ± 14.5	NS
FSH (mIU/mL)	6.9 ± 3.1	7.2 ± 3.6	7.1 ± 2.0	6.7 ± 1.6	NS
LH (mIU/mL)	5.2 ± 3.0	6.5 ± 4.4	4.2 ± 2.7	6.6 ± 4.0	NS
AMH (ng/mL)	2.3 ± 1.4	2.3 ± 1.9	2.6 ± 1.6	1.6 ± 1.0	NS
CA-125 (U/mL)	37.4 ± 21.0	43.5 ± 27.3	46.2 ± 28.2	37.2 ± 15.0	NS

*BMI* body mass index, *E2* serum estradiol, *FSH* follicle-stimulating hormone, *LH* luteinizing hormone, *AMH* anti-Müllerian hormone, *CA-125* cancer antigen 125, *NS* not significant (*p* > 0.05)

^a^Analysis of variance (ANOVA) with repeated measures

As for distribution of causes of infertility in our patient groups, 412 (49.7 %) patients had primary infertility, 249 (30.0 %) patients had secondary infertility, and the remainder had no infertile problems (20.4 %).

## Discussion

According to our analysis, more than half of the endometriosis patients in this study (133/209, 63.6 %) had previously received ovarian surgery before they visited our out-patient department. Therefore, the effect of previous ovarian surgery on their ovarian reserve was worthy to be investigated.

There was a noticeable trend of decreasing serum AMH from control group to endometriosis group. Lower serum AMH level was shown in endometriosis group with previous ovarian surgery, as compared with the endometriosis group without ovarian surgery, although it didn’t reach a statistical significance. It reflected that the attenuation of ovarian reserve after ovarian surgery should not be underestimated, especially in patients with endometriosis. This result supported our hypothesis that superimposed ovarian damage from previous surgery on pre-existing or recurrent endometriosis caused most remarkable deterioration of ovarian reserve.

Such as what had been well-recognized in several studies, we also found that previous ovarian surgery or endometriosis moderately decreased ovarian reserve, respectively [6–12]. Although there was no significant difference of serum AMH level between these two groups in this study.

Prior studies have analyzed pre- and post-operative serum AMH levels in patients with endometriosis [12–29]. However, these studies have been limited by the small number of patients, and definite conclusions have been difficult to make due to different surgeons and procedures [31]. Our study was closer to real world consideration, included the patients from the out-patient department, and enrolled more patient numbers than previous studies, no matter what ovarian surgeries were performed at different hospitals, by different surgeons and with different procedures. Our finding may suggest that previous ovarian surgery exacerbates the depletion of ovarian reserve in the patients with endometriosis regardless of inter-surgeon variability. The impact of ovarian surgery on ovarian reserve in patients with endometriosis should not be neglected. Moreover, it strengthens the findings of prior studies that serum AMH level decreases immediately after ovarian surgery and was continuously lower in serial follow-up [12–26].

It has been known that elevated serum estradiol in early follicular phase predicts poor ovarian response when undergoing in vitro fertilization [35–37]. In this study, we found that the serum estradiol was significantly highest in endometriosis group without previous ovarian surgery and lowest in control group with previous ovarian surgery. It denotes that the patients had endometriosis without any management would have poor ovarian response during in vitro fertilization. Furthermore, patients who received previous ovarian surgery for benign ovarian diseases other than endometriosis, would improve their ovarian response when undergoing in vitro fertilization.

Besides, the significant higher CA-125 level in all endometriosis groups than control groups supports the application of CA-125 in the clinical diagnosis of patients with endometriosis [38, 39].

Prior studies have reported that the existence of endometrioma would attenuate pre-operative serum AMH [8, 10, 12], and the post-operative serum AMH significantly decreased in women with prior endometrioma surgery [11, 14, 17–21, 26]. Different operative procedures also affected the ovarian reserve [15, 23–25, 34]. However, as for patients with endometriosis in this study, the impacts of diagnosis of endometrioma and different operative procedures on serum AMH level were not significant. The findings were consistent with some studies that mentioned the post-operative decrease of ovarian reserve was not significant at the end of three-month follow-up and also limited in experienced surgeons’ hands [27–30, 40]. Therefore, the possible explanation would be that the larger pooled patient number and longer post-operative period (ranging from three to twenty-four months, and the median period was ten months) diminished the influence from endometrioma and different operative procedures.

The finding of our study is very useful for evaluating a patient coming to clinic for infertility counselling. A clinician should always consider impact of previous surgery on current condition of ovarian reserve, especially in those patients with diagnoses of endometriosis [41]. Very low serum AMH level on biochemistry implies further surgical intervention may not be the first choice for an infertile endometriosis patient, under the consideration of insufficient ovarian reserve [42–44].

In the current studies investigating the relationship between ovarian reserve and endometriosis, more and more clinical evidence proved that the post-operative deteriorating of ovarian reserve was remarkable [12, 24–26]. The trend of treatment of endometriosis was gradually towards conservative and individualized, especially for the patients with fertile desire [42–44]. Our findings highlighted the impact of previous ovarian surgeries on ovarian reserve in patients with endometriosis at the out-patient department, despite different operative procedures and inter-surgeon variability. It emphasized the current attitude toward the management of endometriosis with a new perspective from the out-patient department.

Nevertheless, these findings should be interpreted with caution because the impact of the different operative procedures, lesions, and surgeons on ovarian reserve may have ranged from mild to severe. The decreases in serum AMH level also varied considerably. When explaining the risk of surgery on ovarian reserve to patients, it must be done on an individual basis to take their clinical condition into account [44].

There are several limitations to this study. First, the interval from previous ovarian surgery to obtaining the level of serum AMH varied, and it is known that the level of serum AMH changes during the post-operative period from 1 week to 9 months [13–23, 27–29]. We checked the serum AMH level on the patient’s visiting the out-patient department, however the interval from visiting to their previous ovarian surgery ranged from three to twenty-four months, and this may have affected our results. Second, data on the staging and revised American Fertility Society (r-AFS) classification [45] of endometriosis in our patients were incomplete, because some patients’ surgical records from other hospitals had not been obtained at that time. Relevant data was insufficient to evaluate previous surgical impact on ovarian reserve in different endometriosis staging.

Further studies are warranted to investigate the impact of previous surgery on the outcomes of assisted reproductive technology in patients with endometriosis. And further analysis of the impact of previous ovarian surgery on ovarian reserve in patients with different endometriosis staging, as well as the association between the change of serum AMH and benign ovarian diseases other than endometriosis will be further elucidated in the future.

## Conclusions

History of endometriosis and previous ovarian surgery offer clinicians useful information regarding ovarian reserve. Performing repeated ovarian surgery in patients with recurrent endometriosis needs careful consideration and adequate patient counselling because of the predictable deteriorating ovarian reserve.
